# Sensory cue-combination in the context of newly learned categories

**DOI:** 10.1038/s41598-017-11341-7

**Published:** 2017-09-07

**Authors:** Kaitlyn R. Bankieris, Vikranth Rao Bejjanki, Richard N. Aslin

**Affiliations:** 1 Department of Brain and Cognitive Sciences, University of Rochester, Rochester, USA; 2 Department of Psychology, Hamilton College, Clinton, USA

## Abstract

A large body of prior research has evaluated how humans combine multiple sources of information pertaining to stimuli drawn from continuous dimensions, such as distance or size. These prior studies have repeatedly demonstrated that in these circumstances humans integrate cues in a near-optimal fashion, weighting cues according to their reliability. However, most of our interactions with sensory information are in the context of categories such as objects and phonemes, thereby requiring a solution to the cue combination problem by mapping sensory estimates from continuous dimensions onto task-relevant categories. Previous studies have examined cue combination with natural categories (e.g., phonemes), providing *qualitative* evidence that human observers utilize information about the distributional properties of task-relevant categories, in addition to sensory information, in such categorical cue combination tasks. In the present study, we created and taught human participants novel audiovisual categories, thus allowing us to *quantitatively* evaluate participants’ integration of sensory and categorical information. Comparing participant behavior to the predictions of a statistically optimal observer that ideally combines all available sources of information, we provide the first evidence, to our knowledge, that human observers combine sensory and category information in a statistically optimal manner.

## Introduction

Objects and events in the natural environment provide observers with multiple sources of sensory information, within and across several modalities. In order to construct a coherent percept of this multisensory world, we must integrate these sources of information in an efficient manner. For example, the simple act of precisely locating your morning beverage as it dispenses into your mug requires the integration of auditory and visual cues to spatial location. If sensory perception were perfectly accurate, this integration would be trivial because the auditory and visual cues to spatial location would be identical. In reality, however, sensory signals contain uncertainty (potentially due to processing inefficiencies within each sensory modality or noise in the environment) and thus do not provide perfect estimates for the stimulus of interest^1–3^. The imprecise nature of sensory signals can give rise to conflicting cue estimates, which makes the task of cue integration a difficult problem. Our successful interaction with objects in the world indicates that we capably solve this problem on a daily basis, but what are the mechanisms that underlie this process of multisensory integration?

A large body of prior research has evaluated the mechanism of sensory cue-combination across continuous dimensions such as size (e.g., ref. 1). In order to arrive at the most precise estimate of an object’s location, for instance, prior theories have argued that all available sources of information should be linearly combined in a statistically efficient manner, with each cue being weighted according to its reliability (e.g., refs 2 and 4). Applied to the morning beverage example, this approach predicts that humans should weight visual cues to the mug’s location more heavily than auditory cues, because the human visual system more reliably determines spatial location than the human auditory system. Moreover, if this same localization task was performed in the dark when visual information is degraded, we would expect visual cues to be down-weighted compared to their weights in ample light. Formally, we can represent the information provided by an individual sensory signal *A* about a stimulus *S* in the world as a likelihood function, *p*(*A|S*). The value of *S* that maximizes this likelihood function can be thought of as the estimate of *S* suggested by *A*, ${\hat{S}}_{A}$. Given two sensory stimuli *A* and *B* that are conditionally independent (e.g., the sensory uncertainty associated with each modality is independent), the information provided by the combination of both the cues can be written as *p*(*A*,*B|S*) = *p*(*A|S*)*p*(*B|S*). With the assumption that the individual cue likelihood functions are Gaussian, the peak of the combined likelihood function can be written as a weighted average of the peaks of the individual likelihood functions. Formally, the combined estimate of the stimulus is a weighted linear combination of the estimates suggested by the two sensory signals:

1 $$\hat{S}={w}_{A}{\hat{S}}_{A}+{w}_{B}{\hat{S}}_{B}$$

where

2 $${w}_{A}=\frac{\frac{1}{{\sigma }_{A}^{2}}}{\frac{1}{{\sigma }_{A}^{2}}+\frac{1}{{\sigma }_{B}^{2}}}\,{\rm{and}}\,{w}_{B}=\frac{\frac{1}{{\sigma }_{B}^{2}}}{\frac{1}{{\sigma }_{A}^{2}}+\frac{1}{{\sigma }_{B}^{2}}}$$

and σ^2^_A_ and σ^2^_B_ are the variances of *p*(*A|S*) and *p*(*B|S*), respectively. The variance of the combined likelihood *p*(*A*,*B|S*) is given by:

3 $${\sigma }_{AB}^{2}=\frac{{\sigma }_{A}^{2}{\sigma }_{B}^{2}}{{\sigma }_{A}^{2}+{\sigma }_{B}^{2}}.$$

These equations (1–3) describe the behaviour of an ideal observer when combining two cues lying along *continuous* dimensions for a given sensory stimulus, such as spatial location or size, because this approach minimizes the variance of the resulting estimate^5^. Studies evaluating cue combination across continuous dimensions have demonstrated that humans do indeed integrate multiple sources of information efficiently, following this statistically optimal strategy of weighting sensory cues based on their reliability (e.g., refs 2, 5–12).

Sensory cues, however, are not the only source of information relevant to cue-combination. In our natural environment, continuous sensory dimensions are often used to form categorical dimensions and ultimately abstract semantic dimensions^13, 14^. A key question, then, is how we integrate information when noisy estimates from continuous sensory dimensions need to be mapped onto task-relevant categories. For instance, perhaps after you locate your morning beverage, you wish to determine whether it is coffee or black tea. This categorisation task not only requires combining multiple sensory estimates (e.g., colour and smell) but also necessitates mapping these sensory estimates onto the task-relevant categories of coffee and black tea. Accordingly, the weights given to each sensory estimate should be influenced not only by the sensory noise (as in continuous cue integration) in each cue, but also by the statistical properties of the task-relevant categories. In the coffee versus black tea example, you will likely want to rely more on smell than colour to categorise your beverage because the two beverages have less similar smells than colours (i.e., the category means are further separated along the smell dimension than along the colour dimension in terms of just noticeable differences; JNDs). If, on the other hand, you were deciding between a cup of coffee and a latte, you should give a higher weight to the colour cue than the smell cue because coffee and lattes have similar smells but have quite different colours. In other words, in such scenarios the precise distributional properties of the task-relevant categories should influence the cue combination process (e.g., ref. 15). Specifically, the means and variances (assuming Gaussian distributions) of categories, in addition to sensory reliability, should impact how cues are combined in this categorical framework, which is pervasive in our day-to-day lives. In this paper, we therefore seek to bridge the gap between the cue integration literature – which examines how cues with sensory variance are combined – and an extensive body of categorisation literature which investigates how features (with negligible sensory noise) are combined under category uncertainty (e.g., refs 16 and 17).

**Figure 1:**
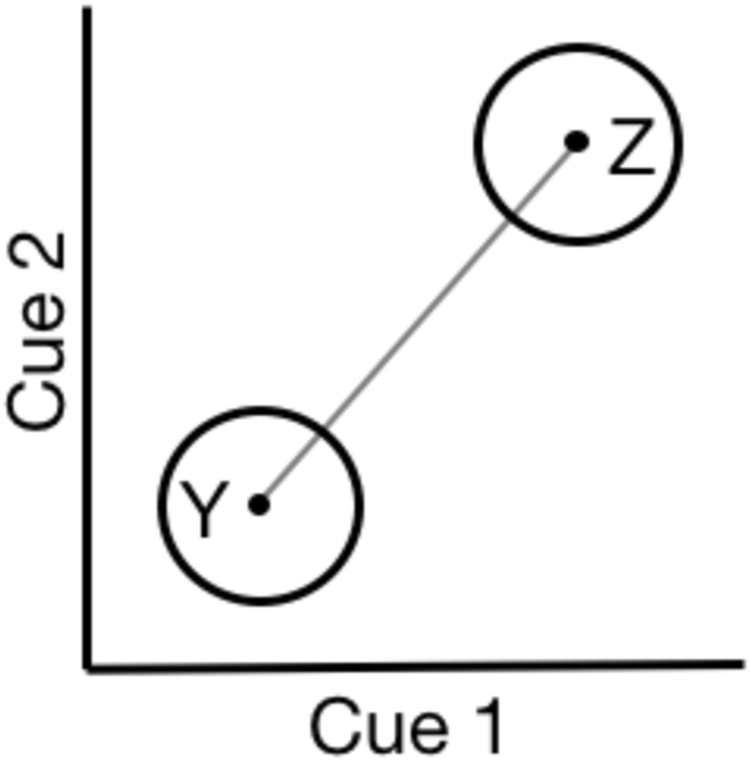
Cue combination involving categories. A depiction of the problem where each category is defined by two cues. The x and y axes represent the strength of each sensory cue. The circles labelled Y and Z represent the mean and covariance of each cue for categories Y and Z for a given participant, under the assumption that the two cues are conditionally independent. The grey diagonal line represents the linear discriminant vector *D* onto which an optimal categoriser projects the received bi-cue signal (see text).

This complex cue integration problem across categorical dimensions could, in principle, be solved by extension of the continuous cue integration model described above (assuming that the covariance matrices for the two categories are equal). Formally, when categorising a multisensory stimulus and assuming once again that the sensory signals are conditionally independent, an ideal observer constructs a discriminant vector linearly connecting the two categories and projects the stimulus onto this vector^18^ (see Fig. 1). This projection of the stimulus onto the discriminant vector is the decision variable *D*, which determines the categorisation of the stimulus based on some criterion:

4 $$D={w}_{A}{\hat{S}}_{A}+{w}_{B}{\hat{S}}_{B}$$

where the information provided by the two cues is represented by *Ŝ*_*A*_ and *Ŝ*_*B*_. The weights for each cue are then given by:

5 $${w}_{A}=\frac{\frac{{\rm{\Delta }}{\mu }_{A}}{{\sigma }_{A,sense}^{2}+{\sigma }_{A,cat}^{2}}}{\frac{{\rm{\Delta }}{\mu }_{A}}{{\sigma }_{A,sense}^{2}+{\sigma }_{A,cat}^{2}}+\frac{{\rm{\Delta }}{\mu }_{B}}{{\sigma }_{B,sense}^{2}+{\sigma }_{B,cat}^{2}}}\,{\rm{and}}\,{w}_{B}=\frac{\frac{{\rm{\Delta }}{\mu }_{B}}{{\sigma }_{B,sense}^{2}+{\sigma }_{B,cat}^{2}}}{\frac{{\rm{\Delta }}{\mu }_{A}}{{\sigma }_{A,sense}^{2}+{\sigma }_{A,cat}^{2}}+\frac{{\rm{\Delta }}{\mu }_{B}}{{\sigma }_{B,sense}^{2}+{\sigma }_{B,cat}^{2}}}$$

where ${\sigma }_{A,sense}^{2}\,{\rm{and}}\,{\sigma }_{B,sense}^{2}$ are sensory uncertainty variances for the two signals and ${\sigma }_{A,cat}^{2}\,{\rm{and}}\,{\sigma }_{B,cat}^{2}$ are the variances of the distribution of the sensory signals occurring in the categories. Lastly, Δ*μ*_*A*_ and Δ*μ*_*B*_ represent the difference between category means along each cue dimension. Accordingly, the formalization of cue combination in tasks involving categorical dimensions posits that an ideal observer should incorporate not only sensory information, but also the manner in which that sensory information maps onto relevant categories.

Previous research has investigated whether human performance is consistent with the predictions of this ideal model, using real world categories such as phonemes (e.g., refs 15, 18 and 19). Results from these studies suggest that humans use both category mappings and sensory information during cue combination across categorical dimensions - specifically, the fit of the ideal model was better than the fit of a model that only considers sensory information. However, the use of natural categories like phonemes makes it very difficult to conduct a *quantitative* comparison between an ideal learner and human behaviour for two reasons. First, natural categories are highly entrenched (i.e., over learned) by extensive prior experience with category exemplars. Second, individual observers have different experience histories with the distributional properties of category exemplars. Thus, the between-participant specificity and limited plasticity of natural categories serve as impediments to a quantitative evaluation of cue combination in the context of categories.

In the present study, we investigated humans’ ability to integrate sensory cues and category information in an efficient manner by teaching participants novel audiovisual categories. Crucially, we manipulated participants’ exposure to exemplars from these novel categories, thereby giving us precise control over their knowledge of the mapping between sensory dimensions and category identity (referred to as “category information” in the rest of the paper). After participants successfully learned these multisensory categories, we tested their cue integration with new exemplars and compared their performance to predictions from a categorical cue integration model that took into account the mapping between the sensory dimensions and category identity. We found that participants’ weights were indistinguishable from the predictions of the statistically optimal categorical cue integration model. Thus, our findings represent the first quantitative evidence, to our knowledge, that participants optimally use both sensory and category information during a cue integration task involving newly learned categories.

## Results

**Figure 2:**
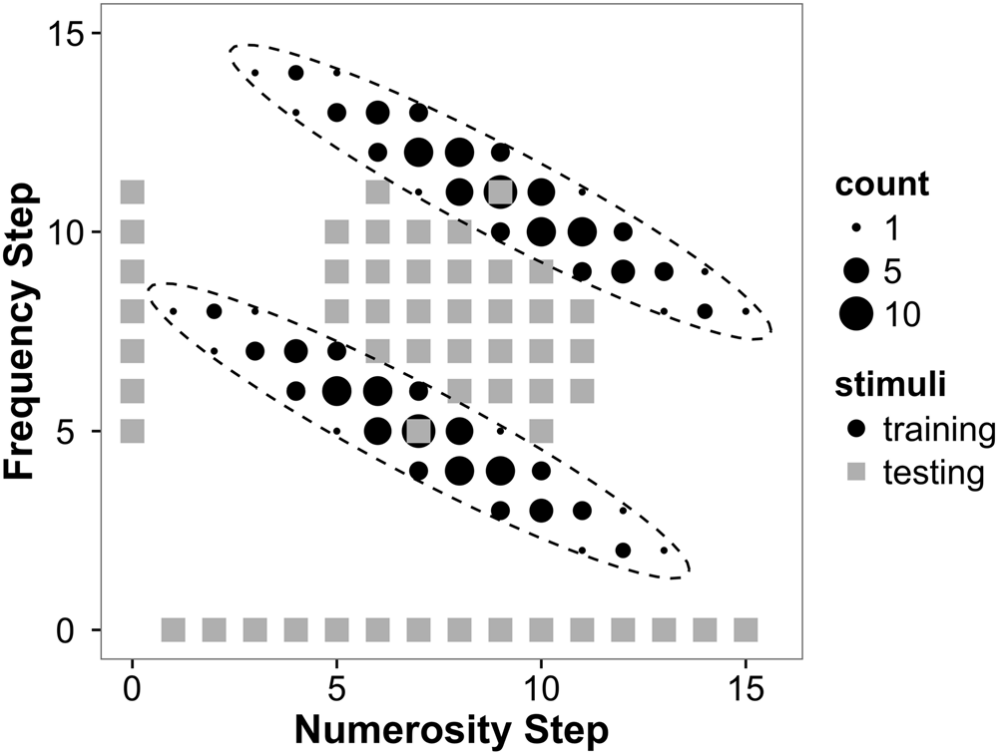
Training and Testing stimuli. Black circles represent the occurrence of exemplars of the 2-cue stimuli during training. The elliptical clusters of black symbols represent the Gaussian distributions of the two task-relevant categories. The size of each symbol represents the number of exemplars of each stimulus that were presented during one learning block. Grey squares represent testing stimuli (bimodal in center, unimodal along the x- and y-axes). Twenty-five repetitions of each testing stimulus were presented. Category labels (taygoo and dohkah) and locations (as below or rotated 90**°**) were counterbalanced across participants.

To quantitatively examine participants’ use of category information during cue combination, we taught participants novel audiovisual categories (labelled ‘taygoo’ and ‘dohkah’), fully controlling the category exemplars to which they were exposed (Fig. 2). The auditory dimension was the pitch (center frequency) of tones in pink noise and the visual dimension was the number of dots in a display area. After participants successfully learned these categories to 90% accuracy, they made category judgments for auditory only, visual only, and audiovisual stimuli (see methods). Crucially, a subset of the audiovisual stimuli presented conflicting cues regarding category membership (i.e., the two component cues differ only slightly in how much they support one category versus the other) in order to estimate auditory and visual weights used by participants during the cue combination process.

**Figure 3:**
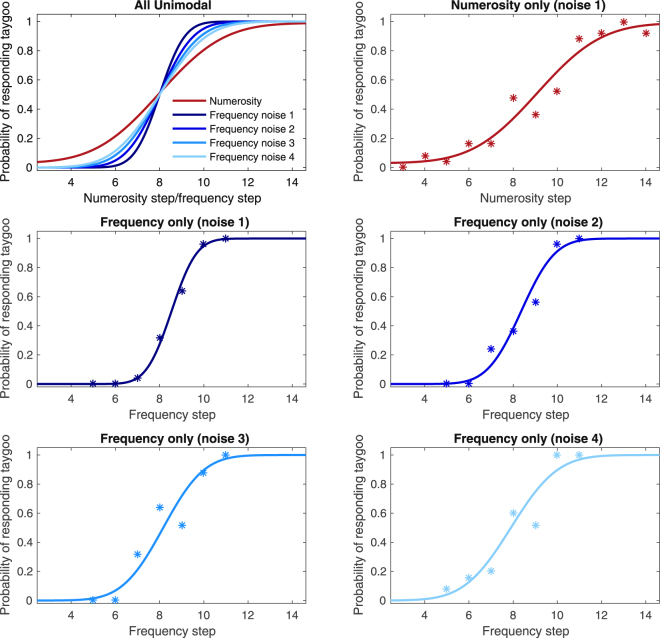
Cumulative Gaussian fits of unimodal trials for a representative participant. The top left panel plots all five unimodal cumulative Gaussian fits with the PSE equalized to allow for easier slope comparison. The remaining panels plot cumulative Gaussian fits along with data for each unimodal condition separately.

**Figure 4:**
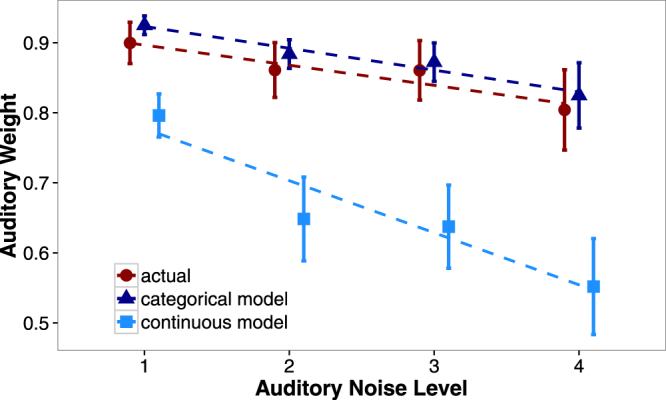
Observed auditory weights for audiovisual trials alongside predictions from the categorical model and the continuous model. Weight predictions for both models are generated including the discount for correlated cues (see main text and equations 6–8). Data points denote means across individual subject weights and error bars denote across-subject standard. Lines are linear fits generated for visualization purposes only.

Before comparing participants’ bimodal behaviour to the statistically optimal categorical model, we fit psychometric functions to characterize their behaviour. First, we estimated unimodal sensory variances (audio and visual) for each participant by fitting psychometric curves to their categorisation performance in each of the five unimodal conditions (four noise levels of auditory only and one noise level of visual only). Fitting participants’ unimodal labelling data with cumulative Gaussian distributions (Fig. 3) yielded the point of subject equality (PSE) and variance (slope) associated with the participants’ representation of the sensory information available in each unimodal cue condition. Next, we fit participants’ categorisation data during each of the four audiovisual conditions (noise 1–4) with psychometric curves and simultaneously ascertained the weights that participants actually assigned to each modality (Fig. 4).

After calculating participants’ auditory weights during our categorical cue combination task, we examined the extent to which their behaviour was consistent with the predictions of an ideal observer using both sensory and category information. Before evaluating the predictions of the categorical cue combination model, it is important to note that the basic model described in the Introduction (Equations 4–5) assumes conditionally independent cues (as shown in Fig. 1). The audio and visual cues used in this experiment, however, are correlated within each task-relevant category (i.e., the cluster of exemplars that define the two Gaussian categorical distributions are tilted, see Fig. 2). Accordingly, in all of our analyses, we expanded the basic categorical cue combination model, so that the calculation of ideal weights accounted for this correlation^20^. Given that the reliability of a given cue in the categorical regime can be written as:

6 $${r}_{i}=\frac{{\rm{\Delta }}{\mu }_{i}}{{\sigma }_{i,sense}^{2}+\,{\sigma }_{i,cat}^{2}},$$

the discounted reliability due to the correlation between cues is

7 $$r{^{\prime} }_{i}={r}_{i}-\rho \sqrt{{r}_{A}{r}_{B}},i={\rm{A}},{\rm{B}}$$

where *ρ* represents the correlation between the two cues from the participant’s point of view. In principle *ρ* is influenced not only by the correlation within each category, but also by the amount of sensory noise on each trial. It is worth noting that given our experimental design, we cannot independently estimate the manner in which sensory noise affects the correlation from the perspective of individual participants. However, we argue that the correlation from a participant’s perspective is dominated in our task by the correlation within each category and thus is *effectively* approximated by it. In support of this argument, we ran several simulations with *ρ* ranging from 0.24 (the correlation within each category) to 0 (the value it would take on if sensory noise completely negated category correlation), and found almost no influence on the predicted weights (see Supplementary Figure 1). Accordingly, in computing the ideal weights, we use the correlation within a category as an effective approximation for *ρ*. The ideal weights are:

8 $${w}_{A}=\frac{r{^{\prime} }_{A}}{r{^{\prime} }_{A}+r{^{\prime} }_{B}}\,{\rm{and}}\,{w}_{B}=\frac{r{^{\prime} }_{B}}{r{^{\prime} }_{A}+r{^{\prime} }_{B}}.$$

Note that in the above formalism, if the correlation is zero, then equation 8 reduces to equation 5.

As a comparison, we also generated predictions from the continuous model (with the discounted reliability due to correlated cues included), which considers only sensory uncertainty. Consider a qualitative description of what one would expect if only sensory uncertainty, and not category uncertainty, were used in making category judgments. As sensory uncertainty is added to the auditory cue, the weight assigned to that noisy auditory cue should decrease (and the weight assigned to the visual cue should show a complementary increase). However, if category information, in addition to sensory information, plays a role in category judgments, then we should see two patterns in participants’ data:

1. *Auditory weights should be higher than those predicted by the continuous model*. Use of category information predicts this pattern of data because the category means are further apart in the auditory dimension than the visual dimension and the individual categories vary less along the auditory dimension than the visual dimension.
2. *The rate at which auditory weights decreases as a function of auditory noise should be slower than predicted by the continuous model*. This prediction also arises from the use of category distributions because the category information is constant across noise level (provided there is sufficient sensory information to support a category judgment).

### Prediction 1: Higher auditory weights compared to the continuous model

If participants are considering category information while performing this cue integration task, their auditory weights should align with the predictions of the categorical model, which are higher than those of the continuous model. While the continuous model uses only sensory information to determine auditory weights, our participants (and the categorical model) have access to the distributional information of the categories. The fact that the category means have a greater distance between them along the auditory frequency dimension makes auditory cues more informative than visual cues at the category level. Likewise, there is less variance for auditory frequency versus visual numerosity within a category, again making auditory information more reliable than visual information at the category level. To test whether participants used this category information, we fit a mixed-effects linear regression predicting auditory weights from weight type (actual, categorical model predictions, continuous model predictions) and noise level (1–4) with a full random effects structure (i.e., random intercepts and slopes per participant). With participants’ observed weights as the reference level (i.e., coded as 0), the beta coefficients for the two other levels of weight type (categorical model predictions, continuous model predictions) indicate whether or not the participants’ weights differ from each of these model predictions. Our analysis found that participants’ actual auditory weights were significantly higher than the continuous model’s predictions (*ß* = −0.08, *SE* = 0.04, *p* < 0.05) but did not differ from the categorical model’s predictions; *ß* = 0.03, *SE* = 0.04, *ns*. These results demonstrate that participants’ auditory weights did not align with the predictions of a model using only sensory information, and were quantitatively indistinguishable from the predictions of a model that incorporates both category and sensory information during cue combination. This finding supports the notion that human observers are sensitive to the distributions of categories during cue combination in such categorical tasks.

### Prediction 2: Smaller effect of noise on the auditory weights

The second unique prediction made by the categorical model of cue combination is that auditory weights should decrease more slowly, across the four noise levels, than predicted by the continuous model. That is, the effect of noise on auditory weights should be smaller if participants are using category information in addition to sensory information. This prediction arises because in addition to sensory information, the categorical model utilizes information regarding the category distributions, which does not change as a function of noise (provided that there is sufficient sensory information available to infer the relevant category). If participants used category information in an ideal manner during this task, their auditory weights should align with the predictions of the categorical model and not the continuous model. Using the mixed-effects linear regression described above, we investigated the rate at which participants’ auditory weights decreased as a function of noise. With participants’ observed weights as the reference level (i.e., coded as 0), the beta coefficients for the interaction of noise level (1–4) and the two other levels of weight type (categorical model predictions, continuous model predictions) indicate whether or not the noise effect for participants’ weights differs from each of these models’ predictions. This analysis revealed that participants’ auditory weights decreased as a function of noise at a rate significantly different from the continuous model’s predictions (*ß* = −0.05, *SE* = 0.02, *p* < 0.05) but indistinguishable from the categorical model’s predictions; *ß* = 0.00, *SE* = 0.02, *ns*. These results demonstrate that the rate at which participants down-weighted auditory information across the noise levels is better predicted by the categorical model than the continuous model. Participants did not use sensory variance as the sole factor influencing their cue weights, but additionally integrated the information provided by category structure into their cue weights. Taken together, our findings represent the first set of evidence demonstrating that humans quantitatively integrate both sensory and category information during cue combination across categorical dimensions in a manner consistent with statistically-optimal behaviour.

## Discussion

In the present study, we examined the computational principles underlying cue combination in a domain that involves multidimensional categories by quantitatively analysing human behaviour during a categorisation task with novel audiovisual stimuli. Critically, a statistically optimal model of cue integration over categorical variables predicts that the distributional properties of the categories themselves (specifically, separation of categories along each cue dimension and category variance along each cue dimension) in addition to sensory variability should influence cue weighting. In contrast to natural (i.e., over-learned) categories for which it is very difficult to estimate the internal knowledge of the category distributions for any given participant, creating novel audiovisual categories allows us to have strict control over participants’ exposure to the stimulus exemplars that define these categories. Thus, we were able to quantitatively compare human behaviour to that of an ideal observer who utilizes both category and sensory information. Our results extend many prior findings in the literature examining cue integration along continuous dimensions, demonstrating that humans appropriately weight sensory cues as a function of their reliability. We find that this principle also holds true when the cues to be combined lie along a categorical dimension, extending the findings of Bejjanki *et al*.^18^ from natural speech categories to recently learned artificial categories. Furthermore, for the first time to our knowledge we demonstrate the optimal use of *category* information (i.e., separation and variance) during a cue combination task by demonstrating that human behaviour quantitatively and qualitatively matched a statistically optimal observer that is sensitive to these sources of information. It is also important to note that previous models of cue combination in the categorisation literature did not incorporate the role of sensory variance because category information was always suprathreshold.

In future research, this model could be used to investigate the properties of cues that yield statistically-optimal cue combination as well as the development of this ability. While it is possible that learners can combine any set of cues in this categorical manner, causal inference likely plays a role in which cues are considered relevant to category membership (e.g., refs 10 and 21). Since we counterbalanced the relationship between the cues across participants (i.e., some participants learned that the category *taygoo* was defined by low pitch and small numerosity while others learned the same category was defined by low pitch and large numerosity), our results suggest that these statistically-optimal principles span some variation in cue relationships. However, further research is needed to determine what limitations exist for the sets of cues and their relationships that can be combined in this manner. Additionally, adapting this paradigm to a developmental context would allow for an examination of how ideal categorical cue-integration develops (cf., ref. 22). Given that children have less experience with the world, it is possible that the cues they are willing to combine in a categorical manner differ from those that adults consider viable for categorical cue combination. Relatedly, examining categorical cue combination in children may allow natural categories to be investigated in a way that is not possible with adult’s entrenched categories. Thus, the present study not only demonstrates quantitatively ideal use of both sensory and category information for cue combination in adults, but also opens up several avenues for future research with children and other special populations.

## Methods

### Participants

Our analysed dataset includes fifteen naïve participants with no known hearing problems and normal or corrected-to-normal vision who were recruited from the Rochester area and compensated $10/hour for their participation. One additional participant was dropped from the final sample because he failed to learn the novel categories to criterion (see below) within four training blocks. Another participant was excluded from group analyses because his performance for unimodal auditory trials at the highest noise level was indistinguishable from chance across all auditory steps and was thus unable to be fit with a psychometric function. Participants were tested individually in a quiet room over a span of four sessions on consecutive days, with each session lasting approximately one hour. Ethical approval was obtained from the University of Rochester Research Subjects Review Board and methods were carried out according to their guidelines and regulations (including obtaining informed consent from all participants).

### Stimuli

We created novel categories defined by two cues: number of visual dots and auditory pitch (see Fig. 2). The number of dots ranged from 11 to 47 in 15 equally discriminable steps. These steps fall along a mathematically logarithmic scale because number is perceived according to Weber’s law. As seen in Fig. 4, black dots were positioned pseudorandomly within a predefined square area to create a specific level of numerosity with no dot overlap. Pitch stimuli were pure tones with frequency ranging from 264 Hz to 502 Hz in 15 equally discriminable steps (again, along a logarithmic scale). We created three additional noise levels for these auditory stimuli by adding pink noise (a signal in which power is inversely proportional to the frequency of the signal: 1/*f*) to the pure tones in increasing percentages. Noise level 1 stimuli were 100% pure tones with 0% pink noise added; noise levels 2–4 were composed of pure tones with 83.3%, 93.8%, and 96.8% pink noise added, respectively, and normalized for overall acoustic energy (percentages indicate percent noise of the root-mean-square value). Extensive pilot testing was conducted to carefully select these cues and their parameters so that the steps along each dimension have equal sizes when expressed as JNDs. Novel categories were defined as two-dimensional Gaussian distributions in the auditory-visual space of the two cues (with the frequency of occurrence of each stimulus rounded to integers as depicted in Fig. 2). Importantly, the two categories in this auditory-visual space cannot be separated using only one cue. That is, no horizontal or vertical line drawn in Fig. 2 will separate these two categories, which necessitates the use of both cues for successful categorisation. Half of the participants learned the categories depicted in Fig. 2 (small number and low pitch, large number and high pitch) and the other half learned these categories rotated 90 degrees (small number and high pitch, large number and low pitch).

### Procedure

Participants were told that scientists had just discovered two new species and their task was twofold: (1) to become an expert at classifying samples and (2) to help the scientists categorise unclassified samples. We informed participants that the two species, labelled with the nonsense words *taygoo* and *dohkah*, could be discriminated using both the pitch of their calls (i.e., auditory frequency) and the number of droppings they produce (i.e., number of visual dots).

### Training

**Figure 5:**
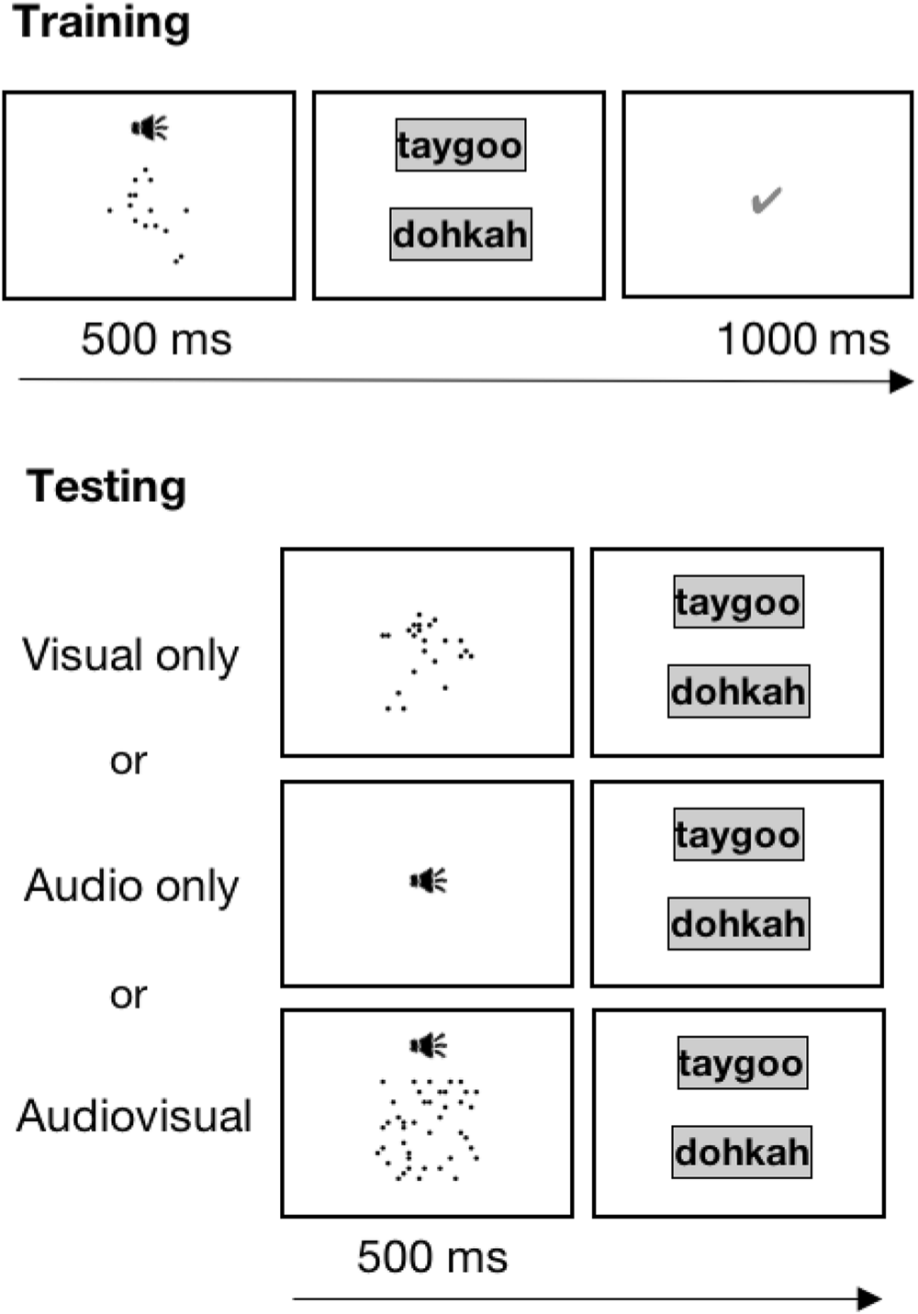
Trial structure. *Training*: example of audiovisual training trials with feedback. *Testing*: example of visual only, audio only, and audiovisual testing trials without feedback.

Each of the four sessions began with a training phase composed of a variable number of blocks, depending on each participant’s learning rate. Each training block presented the full distribution of audiovisual category stimuli (103 of *taygoo* and 103 of *dohkah*) to ensure that all participants experienced the same category statistics. Participants completed as many blocks as necessary to reach 90 percent classification accuracy, with a maximum of four training blocks. As seen in Fig. 5, each trial within a training phase block presented an audiovisual stimulus for 500 ms drawn without replacement from the two-dimensional Gaussian category distributions (see Fig. 2). Two buttons labelled ‘taygoo’ and ‘dohkah’ then appeared on the touch screen and participants touched a button to submit their classification. Feedback indicating whether their choice was correct or incorrect was displayed on the screen for 1000 ms before the next trial began. Category and button labels were counterbalanced across participants.

### Testing

After 90 percent classification accuracy was reached, participants progressed to the testing phase. Audio only, visual only, and audiovisual trials were included in the test phase (Fig. 5). Eight blocks of approximately 130 testing trials were completed during each session – six blocks presenting audiovisual stimuli and two blocks presenting audio only and visual only trials intermixed. The order of these blocks (unimodal or bimodal first) was counterbalanced within participant across day. Each test trial displayed a visual stimulus (or a speaker icon in the case of audio only trials) for 500 ms while an auditory stimulus of equal length was played for audiovisual and audio only trials. As in the practice trials, participants then selected their answer by touching one of two buttons but did not receive feedback. A blank screen was presented for 500 ms before the next trial began. We concentrated our unimodal auditory test items on the seven steps in the middle of the auditory frequency range (steps 5–11), from the mean of one category to the mean of the other category. Since the purpose of these unimodal trials was to ascertain a full psychometric function for each cue individually, and the difference between category means on the numerosity cue is only two steps, we included all 15 numerosity steps in the visual only trials. The audiovisual trials consisted of 31 unique combinations of audio and visual cues (central grey squares in Fig. 2), some of which introduced slight discrepancies between individual cues. For most audiovisual stimuli, that is, the likelihood that the visual component was a ‘taygoo’ was not equal to the likelihood that the auditory component was a ‘taygoo.’ Introducing such discrepancies (i.e., cue conflicts) is crucial for quantitatively measuring cue weights during the integration process. We presented only small cue conflicts to prevent participants from noticing discrepancies, thus encouraging cue integration^4^. Auditory stimuli in audiovisual trials and audio only trials were presented at four different noise levels randomly interleaved throughout the test phase. We presented 25 repetitions of each of these test stimuli, yielding a total of 4175 test trials across four sessions.

### Data Analysis

To analyse categorisation behaviour, we fit psychometric curves to participants’ labelling performance for each of the nine stimulus conditions (one noise level of visual only trials, four noise levels of auditory only trials, and four noise levels of audiovisual trials). For each unique stimulus, the raw response data were organized into arrays specifying the number of trials that a participant responded ‘taygoo’ (out of 25 repetitions). Realizing that individual naïve participants’ data did not always span the entire range from 0.0 to 1.0, we used modified cumulative Gaussian psychometric functions including lapse rates to model their behaviour more accurately^23^. This psychometric function modelled the probability of selecting the category ‘taygoo’ as a mixture of an underlying Gaussian discrimination process and a random guessing process. We coded participant responses as *y*_*i*_ = 0 for a response of ‘dohkah’ and *y*_*i*_ = 1 for a response of ‘taygoo’. We used the following psychometric model:

9 $$\begin{array}{rcl}p({y}_{i}=1|{x}_{i}) & = & \gamma +(1-\gamma -\lambda ){\rm{\Phi }}({x}_{i};\mu ,\sigma )\\ p({y}_{i}=0|{x}_{i}) & = & 1-p({y}_{i}=1|{x}_{i})\end{array}$$

where ${y}_{i}$ is the participant’s categorisation of stimulus ${x}_{i}$ on trial $i$. $\mu$ and $\sigma$ are the mean and standard deviation of the cumulative Gaussian, respectively. For the current task, $\mu$ represents the Point of Subjective Equality (PSE) between the two categories, and $\sigma$ represents the discrimination threshold. Lapse rate parameters are represented by $\gamma$ and $\lambda$, where $\,\gamma$ is the base rate of responding ‘taygoo’ when there is no evidence for category ‘taygoo’, and $\lambda$ is the miss rate, i.e., the probability of responding incorrectly regardless of the amount of information for category ‘taygoo’. We constrained the lapse parameters to be between 0.0 and 0.25, held them constant across noise levels within a condition (audio only, video only, or audiovisual), but allowed them to vary across conditions. Allowing lapse rates to vary across this range accommodates the possibility that our naïve participants may have a high degree of uncertainty for some of the stimuli (compared to highly trained psychophysical observers), without unduly biasing the slope and PSE parameters of the psychometric curve. We used maximum likelihood functions to estimate the parameters of participants’ psychometric functions.

#### Estimating parameters for unimodal performance

The visual only stimuli were presented with no added noise (level 1 only). Accordingly, the likelihood of a subject making a decision ${Y}_{i}$ on visual only trial $i$, when presented with stimulus ${x}_{i}$ can be written as:

10 $${l}_{i}=[(\gamma +(1-\gamma -\lambda ){\rm{\Phi }}({x}_{i};\mu ,\sigma )){Y}_{i}]+[(1-(\gamma +(1-\gamma -\lambda ){\rm{\Phi }}({x}_{i};\mu ,\sigma )))(1-{Y}_{i})]$$

The likelihood function for the entire set of visual only data for a given subject is then:

11 $${L}_{Vis}=\,\prod _{i=1}^{N}{l}_{i}$$

where *N* is the total number of visual only trials.

The audio only trials were presented at four noise levels. Thus, the likelihood of a subject making a decision ${Y}_{i,j}$ on audio only trial $i$ for noise level $j$, when presented with stimulus ${x}_{i,j}$ can be written as:

12 $$\begin{array}{rcl}{l}_{i,j} & = & [(\gamma +(1-\gamma -\lambda ){\rm{\Phi }}({x}_{i,j};{\mu }_{j},{\sigma }_{j})){Y}_{i,j}]\\  &  & +\,[(1-(\gamma +(1-\gamma -\lambda ){\rm{\Phi }}({x}_{i,j};{\mu }_{j},{\sigma }_{j})))\,(1-{Y}_{i,j})]\end{array}$$

The likelihood function for the entire set of audio only trials for a given subject is then given by

13 $${L}_{Aud}=\,\prod _{j=1}^{4}\prod _{i=1}^{N}{l}_{i,j}$$

where *N* is the number of audio only trials for each noise level and there are four noise levels.

#### Estimating parameters for audiovisual performance

During each trial of the audiovisual categorisation task, participants were presented with an audio and visual stimulus simultaneously. Crucially, in a subset of the audiovisual stimuli, there were cue conflicts between the two modalities, which allows for the estimation of the weights that participants used in combining the two cues. Under the linear cue combination assumption, we consider the effective stimulus in this task to be a weighted combination of the two stimuli. Parameters for the psychometric model ($\mu ,\sigma ,\gamma ,{\rm{and}}\,\lambda$) and the weights assigned to each modality (${w}_{a}\,{\rm{and}}\,{w}_{v}$) for bimodal performance were computed from maximum likelihood fits to the raw bimodal performance data for each participant. Specifically, the audiovisual condition had four noise levels, so the likelihood of a subject making a decision ${Y}_{i,j}$ on audiovisual trial $i$ for noise level $j$, where the presented stimulus was ${x}_{{a}_{i,j}}$ in the auditory domain and ${x}_{{v}_{i,j}}$ in the visual domain, can be written as:

14 $$\begin{array}{rcl}{l}_{i,j} & = & [(\gamma +(1-\gamma -\lambda ){\rm{\Phi }}(((1-{w}_{a}){x}_{{v}_{i,j}}+{w}_{a}{x}_{{a}_{i,j}});{\mu }_{j},{\sigma }_{j})){Y}_{i,j}]\\  &  & +\,[(1-(\gamma +(1-\gamma -\lambda ){\rm{\Phi }}(((1-{w}_{a}){x}_{{v}_{i,j}}+{w}_{a}{x}_{{a}_{i,j}});{\mu }_{j},{\sigma }_{j}))(1-{Y}_{i,j})]\end{array}$$

Since ${w}_{a}\,{\rm{and}}\,{w}_{v}$ sum to one, the above expression replaces ${w}_{v}$ with $1-{w}_{a}$. The likelihood function for the entire set of audiovisual trials for a given subject is then given by:

15 $${L}_{AV}=\,\prod _{j=1}^{4}\prod _{i=1}^{N}{l}_{i,j}$$

where *N* is the number of audiovisual trials at each of the four noise levels.

#### Avoiding local maxima when fitting psychometric functions

To avoid converging on local maxima, rather than on the desired global maximum likelihood, we repeated each maximum likelihood fit starting from five randomly chosen initial values for the parameters. We then selected the parameters that corresponded to the fit with the best maximal likelihood value, across the five fitting runs, as the best-fit parameters for the psychometric model.

### Data Availability

The datasets generated during and analysed during the current study are available from the corresponding author on reasonable request.

## Electronic supplementary material

Supplementary Figure 1
