# Supplementary material for: Sensory cue-combination in the context of newly learned categories
Source: Sci Rep. 2017 Sep 7;7:10890. doi: 10.1038/s41598-017-11341-7 (PMC5589839; doi:10.1038/s41598-017-11341-7)
Supplement: Supplementary file 1 — Supplementary Figure 1 [file 41598_2017_11341_MOESM1_ESM.pdf]

Supplementary information

Sensory cue-combination in the context of newly learned categories

Kaitlyn R. Bankieris, Vikranth Rao Bejjanki, Richard N. Aslin

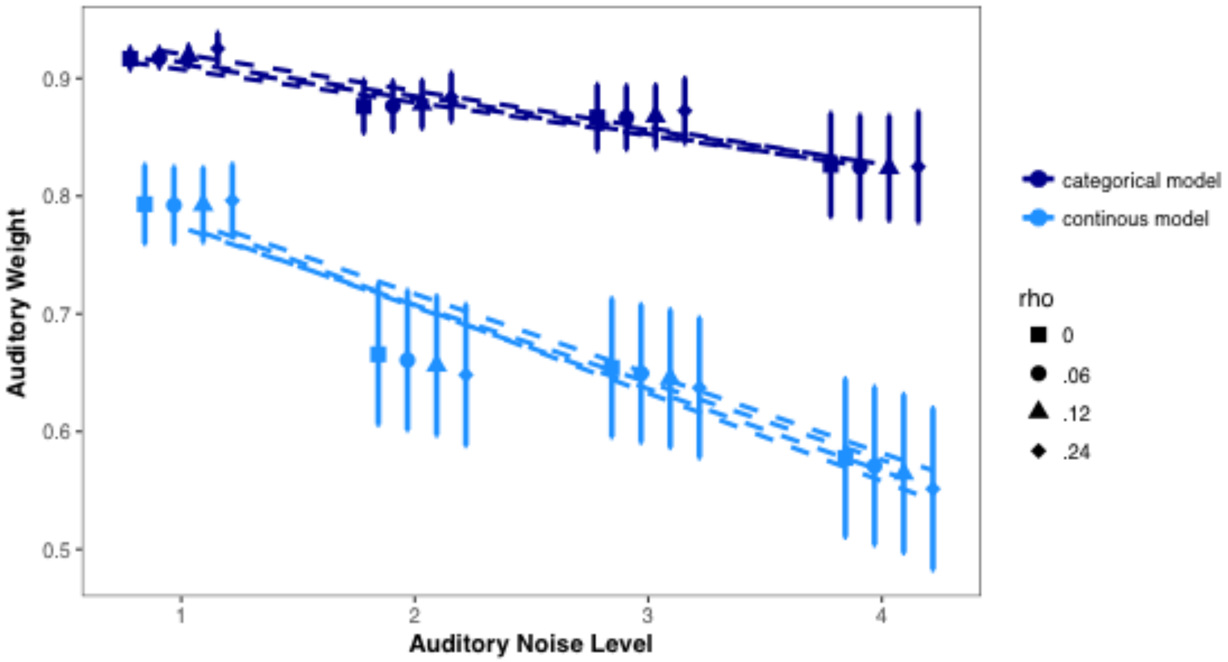

Figure S1: Predicted auditory bimodal weights generated from the categorical and continuous models, accounting for varying levels of cue correlation (rho). Predicted weights for each model are calculated from equations 6-8 with different values of rho as indicated in the legend. Data points denote means across individual subject weights and error bars denote across-subject standard error.
